# Review of guidelines on expression, storage and transport of breast milk for infants in hospital, to guide formulation of such recommendations in Sri Lanka

**DOI:** 10.1186/s12887-018-1244-2

**Published:** 2018-08-14

**Authors:** Ranmali Rodrigo, Lisa H. Amir, Della A. Forster

**Affiliations:** 1 Department of Paediatrics, University of Kelaniya, 6 Thalagolla Road, Ragama, 11010 Sri Lanka; 2 Judith Lumley Centre, La Trobe University, 215 Franklin Street, Melbourne, VIC 3000 Australia; 3 Royal Women’s Hospital, Locked Bag 300, Parkville, VIC 3052 Australia

**Keywords:** Expressed breast milk, Storage, Transport, Preterm infant

## Abstract

**Background:**

Sick newborns in neonatal units who are unable to breastfeed are fed expressed breast milk. In Sri Lanka, most mothers stay in hospital throughout baby’s stay to provide this milk freshly. In other countries mothers go home, express breast milk at home and bring it to hospital. There are concerns about the safety of transported expressed milk if used in a tropical middle-income country. The aim of this paper is to compare and contrast advice offered by different hospitals and organizations on how to express, store and transport breast milk safely.

**Methods:**

We assessed guidelines used by hospital staff of the four Level 3 neonatal units in Melbourne, Australia, National Health Service UK, guidelines and training manuals of the Human Milk Banking Association of North America, the World Health Organization and an information leaflet from Family Health Bureau, Sri Lanka. Information on breast milk expression, storage and transport provided by the guidelines were tabulated under seven topics: general information; container for milk collection; hand expression; using a pump for expression; storage; thawing / warming; and transport of expressed breast milk. The AGREE II tool was used to assess the guidelines written for hospital staff.

**Results:**

There was considerable agreement on most recommendations provided by these sources, but no single source covered all topics in full. Most recommend hand expression as the initial method for expressing of breast milk, followed by breast pump use, except the Sri Lankan recommendations which strongly discourages the use of breast pumps. Durations of storage under various conditions are generally similar in the different recommendations. Most guidelines recommend a ‘cool box’ or container with ice or freezer packs for transportation of milk.

**Conclusion:**

A single document containing recommendations on all aspects of expressing, storing and transporting breast milk should be available for each unit, with the same basic information for mothers and the healthcare staff and further technical details for staff if required. The Sri Lankan recommendations need to be updated based on current worldwide practices and further studies are needed to establish a safe method of transport of expressed breast milk in Sri Lanka.

**Electronic supplementary material:**

The online version of this article (10.1186/s12887-018-1244-2) contains supplementary material, which is available to authorized users.

## Background

Breastfeeding has numerous advantages to the baby and mother including reduced infections and higher intelligence in the breastfed children and reduced breast cancer with lower risk of diabetes for the mothers [1]. Showing all mothers how to ‘.. . maintain lactation even if they should be separated from their infants’ is one of the ten steps to successful breastfeeding, identified under the Baby Friendly Hospital Initiative (BFHI) [2].

A considerable number of babies who are in neonatal units either due to prematurity or other illness are unable to breastfeed directly as they are receiving invasive ventilatory support or are too premature to have coordinated, safe, sucking and swallowing reflexes [3]. These babies need to be provided with expressed breast milk, which can be given to the baby via several different methods including nasogastric or orogastric tubes, cup feeds, and syringe or dropper feeds [4]. The numbers of babies using these different methods have not been published in Sri Lanka or elsewhere. The monthly statistics of the unit that the first author is attached to, reveal that there were 3382 births in 2017, with 744 admissions to the neonatal unit including 61 babies born in other hospitals. This unit is a referral centre for fetal medicine. The number of babies less than 36 weeks gestation who were admitted to the neonatal unit, who would certainly have been given expressed breast milk at some point, was 272, that is 36% of the admitted babies. Some of the other babies more than 36 weeks who were on the ventilator or double phototherapy would also have received expressed breast milk.

In most developed countries like Australia, the United Kingdom and the United States of America, mothers are discharged from hospital even if their infants remain in the neonatal unit. Therefore, if they are providing breast milk for their babies they have to express breast milk at home and bring it to the hospital. In Sri Lanka, most mothers spend the entire time their baby is in the neonatal unit in hospital, and they provide fresh expressed breast milk for each feed. However, with increasingly lower gestation babies surviving in the neonatal units in Sri Lanka, mothers have to spend many weeks in the hospital, which becomes difficult in practice for some mothers. Mothers whose babies are in the neonatal unit do not even get a bed of their own at times due to overcrowding in the postnatal ward; there are situations where several mothers whose babies are in the neonatal unit have had to share a single bed. Meals are provided by the hospital, but most mothers wish to have their meals brought from home by relatives in the belief that lactating mothers should be provided with special home-made meals. A restricted number of relatives are allowed to visit the mother during the three visiting hours per day, but children are not allowed to come to the postnatal ward.

As there are concerns about the safety of using breast milk expressed at home and brought into hospital, this is currently not encouraged, especially from long distances; a safe method of expressing, storing and transporting breast milk for sick newborns in Sri Lanka therefore needs to be established. Currently there is no written feeding guideline for the unit at which the principal author works in Sri Lanka, but the hospital strives to adhere to the 10 steps of BFHI [2] in taking decisions regarding the feeding plan for individual babies. The different modes of feeding have not been formally evaluated or described in a study yet, in Sri Lanka.

A written guidance is used in the unit in assessing fitness for discharge, with the minimum criterion being that the baby is fully breast milk fed, using a combination of breastfeeding and cup-feeding (without use of bottles and teats) and being 1.2 kg by weight (around 34 weeks). The mothers receive intensive support in lactation management during hospital stay and the babies are closely followed-up for weight gain after discharge.

Having access to a refrigerator is essential for breast milk storage if it is to be expressed at home, stored and transported to the hospital later. National Sri Lankan data from 2009/10 which is the latest available, show that 60% of urban households and 38% of rural households have a refrigerator [5]. This number is certainly higher now although no more recent data are available, either for the country or for any particular hospital.

The bacteriological contamination of stored human milk and fresh milk has shown varied results in studies conducted under different conditions leading to differences in recommendations made by different institutes [6–10]. Studies have also examined the biochemical properties of stored milk [11, 12]. As there is currently no gold standard in best practice for expressing, storing and transporting human milk from home to hospital specifically for sick and preterm infants, we set out to review recommendations from a number of sources.

## Methods

In order to establish safe standards for transporting expressed breast milk in Sri Lanka we initially identified information sources from Melbourne, Australia, where the researchers had access to the detailed protocols and guidelines of the Level 3 neonatal units, and other countries where transportation of expressed breast milk is common practice. The information sources we used are given in Table 1. The resources written for hospital staff were evaluated using the Appraisal of Guidelines for Research & Evaluation – II (AGREE-II) instrument [13] to assess the quality of guidelines. The information sources for which the AGREE II instrument was used has been indicated in Table 1. Guidance given by the Level 3 neonatal units in Melbourne, Australia and recognized health authorities in UK, USA, and Sri Lanka as well as the World Health Organization (WHO) recommendations were used. The documents from Australia, UK and USA are meant for neonatal intensive care unit hospital staff and mothers. The Sri Lankan fact sheet is mostly used in the community, but is given to some mothers with babies in the neonatal unit as a written guidance to the method of expressing breast milk by hand. The WHO recommendations, which are meant for global usage including resource limited settings, are used by the Family Health Bureau of the Ministry of Health, Sri Lanka, for training of all health care personnel in the country on breastfeeding issues.

**Table 1:** List of information sources reviewed

Institute	Year	Names of guidelines / protocols / webpage titles / fact sheets	Audience	AGREE II Instrument [13]
MHW	2013	1. Breastfeeding guide [17]	Mothers	Not used
	2012	2. Breast Milk Expression Procedure [18]	Staff	Used
	2014	3. Breast Milk Expressing Equipment Management Procedure [19]	Staff	Used
	2015	4. Expressed Breast Milk (EBM): Storage and Management in Neonatal Services Procedure [20]	Staff	Used
	2014	5. Expressing breast milk [34]	Mothers	Not used
		6. Cleaning your breast pump equipment [36]	Mothers	Not used
RWH	2015	1. Expressing breast milk for sick or preterm babies [21]	Mothers	Not used
	2013	2. Expressing breast milk [22]	Mothers	Not used
	2011	3. Infant Feeding: Expressed Breast Milk: Management in Newborn Services [37]	Staff	Used
	2008	4. Using a breast pump [35]	Mothers	Not used
Monash	2014	1. Expressed breast milk (EBM) safe management and storage [23]	Mothers	Not used
	2011	2. Expressing breast milk [24]	Mothers	Not used
RCH	2013	1. Breastfeeding a baby in hospital [25]	Mothers	Not used
	2013	2. Breastfeeding at The Royal Children’s Hospital [26]	Mothers	Not used
NHS (UK)	2016	1. Expressing and storing breast milk [27]	Mothers	Not used
	2014	2.Breastfeeding your premature baby [28]	Mothers	Not used
HMBANA	2011	1. Best Practice for Expressing, Storing and Handling Human Milk in Hospitals, Homes, and Child Care Settings.© HMBANA. 3rd Edition [30]	Staff	Used
WHO/UNICEF/ Wellstart	2009	1. Baby-Friendly Hospital Initiative - revised, updated and expanded for integrated care. Section “Results” Breastfeeding Promotion and Support in a Baby-Friendly Hospital. A 20-h course for maternity staff [31]	Staff	Used
SL		How to express breast milk [32]	Mothers	Not used

*MHW* Mercy Hospital for Women, Melbourne, Australia, *RWH* Royal Women’s Hospital, Melbourne, Australia, *Monash* Monash Melbourne, Australia, *RCH* Royal Children’s Hospital, Melbourne, Australia, *NHS* National Health Service, UK webpages, *HMBANA* Human Milk Banking Association of North America, *WHO/UNICEF* World Health Organization / United Nations Children’s Emergency Fund, *SL* Sri Lanka

The guidelines of the National Health and Medical Research Council, Australia and the Academy of Breastfeeding Medicine, USA protocol were not used as these guidelines focus on expressing and storing human milk for healthy term babies when mothers are separated from this infants, e.g. for paid employment [14, 15]. A recent review by Peters et al. provides one of the most comprehensive systematic literature reviews on the safe management of expressed breast milk [16]. However that review did not make a clear distinction between expressing milk for sick preterm babies in hospital and healthy term infants at home [16].

The guidelines for hospital staff and fact sheets for parents provided to mothers from the four hospitals in Melbourne, Australia which have level 3 neonatal units, namely Mercy Hospital for Women (MHW) [17–20], the Royal Women’s Hospital (RWH) [21, 22], Monash Health (MH) [23, 24] and the Royal Children’s Hospital (RCH) [25, 26]; the National Health Service (NHS) website from the United Kingdom [27, 28]; the guideline of the Human Milk Banking Association of North America [29, 30]; information provided in the World Health Organization training course for maternity staff on breastfeeding promotion and support in a baby friendly hospital [31]; and the fact sheet for mothers on breast milk expression published by the Ministry of Health, Sri Lanka [32] were used to identify the recommendations made regarding storage and transport of expressed breast milk for sick babies in hospital. If these information sources provided advice separately for both categories of babies – those in neonatal units and those at home, only those relevant to the hospitalized infants was used. Two of the institutes whose recommendations were reviewed (Mercy Hospital for Women, Melbourne and HMBANA) also provided advice regarding milk being brought in for human milk banking and milk donation, but this information was not considered in the review.

The RCH and RWH recommendations are available online for access by the general public, while the MHW and Monash guidelines are available only on the intranet of each hospital for internal use only. The NHS, UK has a web page accessible by the general public with useful attractive illustrations regarding expression of breast milk, and advises to contact hospital staff regarding storage of milk for sick newborns.

### Guideline quality assessment by the AGREE II instrument

The guidelines written for hospital staff were appraised by two assessors using the AGREE-II instrument. The assessment is done based on 23 items classified into six domains – namely scope and purpose, stakeholder involvement, rigor of development, clarity of presentation, applicability and editorial independence [13]. Each item is scored on a 7-point scale. The scores given by the assessors are presented as percentages based on the maximum possible score for each domain. The maximum possible score depends on the number of assessors and number of items in a particular domain that were assessed. In our assessments all 23 items were scored and none were left out. In some of the documents from Mercy Hospital for Women, stakeholder involvement was unclear and clarifications were made by contacting the staff of the Department of Paediatrics and Human Milk Bank at the hospital.

### Review of the recommendations provided by the information sources

The recommendations provided by the chosen information sources were categorized under the following topics and tabulated.

1. General information on expression of breast milk and preparation for expression (Additional file 1: Table S1)
2. Container for collection and storage of expressed breast milk (Additional file 2: Table S2)
3. Hand expression of breast milk (Additional file 3: Table S3)
4. Using a pump for expression of breast milk (Additional file 4: Table S4)
5. Storage of expressed breast milk (Additional file 5: Table S5)
6. Thawing and warming of stored expressed breast milk (Additional file 6: Table S6)
7. Transport of expressed breast milk (Additional file 7: Table S7)

A detailed section on developing the healthcare workers’ communication skills to counsel and build the self-confidence of mothers is available only in the WHO guidance [31].

## Results

### Assessment of guideline quality using the AGREE II instrument

The percentages obtained for each domain by the six guidelines appraised using the AGREE II instrument are given in Table 2. All six guidelines scored well in the two categories of scope and purpose, and clarity of presentation, but poorly in the category of rigor of development. Editorial independence was also a weak point in most except for the WHO guideline [31]. Applicability of most guidelines, except the WHO guidelines had room for improvement. Overall, the WHO guideline was of the highest quality according to the AGREE II assessment, however the most amount of evidence based information was available in the HMBANA guideline [30].

**Table 2:** Assessment of guideline quality by AGREE II tool

	MHW 2 (%)	MHW 3 (%)	MHW 4 (%)	RWH 3 (%)	HMBANA (%)	WHO (%)
Scope and purpose	97	94	94	89	78	94
Stakeholder involvement	42	61	50	25	22	100
Rigor of development	16	13	13	9	44	56
Clarity and presentation	92	89	89	92	94	100
Applicability	25	58	38	40	29	96
Editorial independence	13	25	25	13	50	100

*MHW 2* Breast milk Expression Procedure (2012) from Mercy Hospital for Women [18], *MHW 3* Breast Milk Expressing Equipment Management Procedure (2014) from Mercy, Hospital for Women [19], *MHW 4* Expressed Breast Milk (EBM): Storage and Management in Neonatal Services, Procedure from Mercy Hospital for Women [20], *RWH 3* Infant Feeding: Expressed Breast Milk: Management in Newborn Services from, Royal Women’s Hospital [37], *HMBANA* Best Practice for Expressing, Storing and Handling Human Milk in Hospitals, Homes, and Child Care Settings.© HMBANA. 3rd Edition from Human Milk Banking Association of North America [30], *WHO* Baby-Friendly Hospital Initiative - revised, updated and expanded for integrated care, Section 3 - Breastfeeding Promotion and Support in a Baby-Friendly Hospital. A 20-hour course for maternity staff from the World Health Organization, UNICEF and Wellstart [31]

### Review of information provided by the information sources

#### General information on expression of breast milk and preparing for expression

The Melbourne hospitals with maternity units, HMBANA and WHO all mentioned the need to commence expression of breast milk as soon as possible after birth with the latter two mentioning that it should ideally be within 6 h of delivery [18, 21, 22, 24, 30, 31]. The Sri Lankan fact sheet and the NHS website did not comment on the timing of commencement of breast milk expression. All guidelines / institutes which provided recommendations on how to initiate breast milk expression suggested hand expression as the initial method. Subsequently they all recommended the pump as the preferred method with the exception of the Sri Lankan fact sheet [32].

The need for regular expression of breast milk – 3 to 4 hourly – along with the need for night time expression was stressed by all guidelines [18, 21, 22, 24, 25, 27, 30–33].

All guidelines advised women to wash their hands before expression, with the NHS, HMBANA and Sri Lankan recommendations being specific about mentioning that soap and water should be used for washing [27, 30, 32]. HMBANA guidelines also advise women to clip nails, remove rings and nail polish and recommended single-use towels for drying the hands after washing [30].

In order to encourage the mother’s let-down reflex and maximize milk output, most of the guidelines advise mothers to be seated, relaxed and observe a photograph of their baby if the expression is being done away from the bedside of the baby [21, 25, 30, 31, 34]. The Sri Lankan fact sheet does not mention these, although use of warm compresses and gentle massage are advised [32].

#### Container for collection and storage of expressed breast milk

There is a mix of recommendations about containers to collect and store milk in, with some recommending only ‘clean’ containers [30–32] while others recommend ‘sterile’ containers [20, 21, 23, 25, 27] . In the first few days after giving birth a mother may have only small amounts of colostrum available. It is easier to collect this small quantity in a syringe. Sterile syringes for colostrum are specifically mentioned by MHW and RWH [20, 21]. Single use sealable plastic containers are mentioned by the RWH and MHW whereas the Sri Lankan fact sheet recommends wide-mouthed containers which should be washed, boiled and reused. As there is no storage involved at present in Sri Lanka; the same container is usually used for cup-feeding the baby [20, 21, 32]. The filling of the container by expressed milk was most often recommended to be restricted to three quarters, by institutes where milk is stored by freezing [20, 21, 30]. MHW, RWH and RCH guidelines refer to the number of expressions that can be included in one container, with a range from two to several within 1 day [20, 21, 25] being recommended. RWH specifies that the fresh milk should be chilled before adding to the already frozen milk in the storage container [21].

The need for clear labelling of expressed milk was mentioned by most guidelines. Most hospitals provide the mother with printed labels which have the baby’s identification details, and also request the mother to write the date and time of expression, with some having labels on which date and time of thawing and additive use can also be mentioned [20, 21, 23].

#### Hand expression of breast milk

The placement of the fingers on the breast for hand expression was explained in slightly different ways. In essence, all the recommendations advise placing the fingers at the edge of the areola / several centimeters back from the nipple with thumb opposite the forefinger / other four fingers to hold the areola in between. The WHO description is complicated as it makes statements like ‘compress the breast over the *ducts*’ and ‘*try* pressing your thumb and fingers back towards your chest’ [31] p.164, rather than providing simple, directed, stepwise statements.

The need to press backwards towards the chest wall prior to compression is mentioned by most. Compression is very basically described as ‘press thumb and 1^st^ finger together’ in the Sri Lankan pamphlet, while the WHO and NHS stress the importance of avoiding sliding or rubbing along the breast to avoid damage to the skin [27, 31, 32]. Most advise to move around areola to express from all ducts. The fact that hand expression of breast milk should not be painful if done correctly is mentioned clearly.

Most information sources advised to express from one breast until the flow slows down and then to switch over to the other breast. The Sri Lankan fact sheet advises to express for 20–25 min, while the MHW guideline says to switch over to the other breast or different site on the same breast when milk flow slows, with no specific duration being given [18, 32]. The RWH guideline mentions clearly that in the first few days the volume would only be a few drops [22].

The Sri Lankan fact sheet explicitly states that pressing or pulling on the nipple or massaging the breast does not result in expression of breast milk [32].

#### Using a pump for expression of breast milk

As mentioned earlier the Sri Lankan fact sheet strongly discourages use of pumps for expression of breast milk [32]. Personal experience has found the reason given for this is the idea, especially among midwives and nurses, that pumps are painful and ineffective as staff have not had much exposure to technologically advanced equipment in this field. There are also concerns regarding hygiene: pumps are thought to be a potential source of harmful bacteria [32]. The MHW guideline advises to hand express prior to use of a pump to stimulate the let-down reflex [18]. Only the MHW and RWH guidelines provided details on how to use an electric breast pump [34, 35]. They advise to place the breast shield centrally over the nipple and to initially use low suction with high speeds and vice versa as the milk starts flowing. Mothers are advised to use single breast pumps for 20–30 min and a double for 10–15 min each time [34]. The RCH guideline advises to seek the assistance of the nursing staff [25].

Clear cleaning instructions are given by the MHW and RWH [35, 36]. HMBANA states that majority of hospitals give each mother a sterile kit but only advises cleaning between use [30]. The MHW advises the same for reusable pump kits, except when the baby is preterm or sick when daily sterilisation is recommended [19]. The NHS advises to sterilise before and after each use [27, 28].

#### Storage of expressed breast milk

Expressed breast milk can be stored under different conditions. The recommended safe time period given by the different guidelines for keeping in room temperature ranges from 4 to 8 h. The ambient room temperature is not mentioned. If fresh milk is to be chilled / frozen RWH mentions that it should be done within 1 h of being expressed, while the MHW states that excess fresh milk placed in the refrigerator for < 48 h can be frozen [18, 20, 37].

The recommended duration of safe storage in a refrigerator for a baby in the hospital ranged from 24 h in the Sri Lankan pamphlet to 2–4 days by HMBANA [30, 32]. The MHW and RWH guidelines recommend 48 h while the RCH requests mothers to bring in the expressed milk, which has been kept in the refrigerator, to the hospital within 24 h of expression, for freezing [20, 21, 25]. The recommendation by the WHO and the Sri Lanka pamphlet for all babies in general, and by MHW specifically for babies in hospital, is that milk should be stored for ≤3 months in the freezer [20, 31, 32]. The RWH guidelines recommend safe frozen storage times of a) 2 weeks or b) 3 months c) 6 months respectively for milk stored in a freezer that is a a) compartment within the refrigerator or b) the freezer has a separate door from that of the refrigerator or c) if the freezer is completely separate without being part of a refrigerator [21]. The RCH guideline does not clearly state the maximum duration that milk for a sick baby can be stored in hospital [25]. The Monash guideline recommends the safe duration of refrigeration for freshly expressed breast milk as being up to 72 h, but does not specify a safe time period for frozen milk [23].

#### Thawing and warming of stored expressed breast milk

The sources mention a wide range of methods to thaw frozen milk and safe usage times following thawing. If thawed in room temperature, the MHW guideline states that it should be used within 12 h or maximum 24 h if placed in the refrigerator immediately after thawing outside the refrigerator [20]. The HMBANA guideline however recommends only a 4 h safety period (until next feed) for the latter method [30]. Others do not mention this method of thawing.

If the expressed breast milk is thawed by placing in the refrigerator (one of the two commonly recommended methods) the WHO guideline recommends 12 h and the RWH guideline recommends 48 h, while the guidelines of MHW [20, 23, 30, 31, 37], Monash and HMBANA recommend 24 h as the safe period for usage. The HMBANA guideline additionally mentions that it should be used within 4 h once placed in room temperature [30].

Thawing by rapid warming using luke warm water was the other method mentioned by the guidelines from MHW, RWH, HMBANA, WHO, and it was the only method mentioned by the RCH guidelines [20, 25, 30, 31, 37]. Of these, the RWH and Monash guidelines said to use within 4 h, while MHW and HMBANA recommended to keep outside the refrigerator only until the end of the feed and mentioned that reusing any remainder that has been separated before the feed commenced is possible within 4 h if placed in refrigerator until then [20, 23, 30, 37]. The WHO recommendation was to use within 1 h [31].

All guidelines stated that there should be no refreezing after thawing. All sources also stated that a microwave should not be used for thawing.

The Sri Lankan fact sheet did not provide any information on methods of thawing or safe usage durations following thawing [32]. This guideline mentioned that refrigerated milk needs to warmed by placing in luke warm water, but advised not to boil or reheat. The latter advice was given by the WHO guideline as well [31]. Refraining from boiling was not mentioned by any other sources, while avoidance of reheating was specifically not mentioned by the MHW, RWH, RCH or HMBANA guidelines [20, 21, 25, 30, 37].

#### Transport of expressed breast milk

The RCH guideline advised not to bring frozen milk in to the hospital and Monash mentioned that frozen milk should be maintained in frozen state for transport while refrigerated should be maintained between 1 and 4 °C [23, 25].

An insulated food container or cool-box was recommended by all the Melbourne hospitals for transport of expressed breast milk. The RWH, Monash and RCH guidelines requested transportation with ice or freezing blocks [21, 23, 25]. The MHW guideline showed preference for gel or cold packs over ice for transport [20]. If the amount of thawed milk was < 25% the MHW guideline advised to place the milk in the freezer while it was to be placed in the refrigerator if the amount thawed was more extensive [20]. The RCH guideline did not encourage mothers to bring in frozen milk and advised mothers to bring in refrigerated milk less than 24 h old to hospital [25]. The RWH guideline recommended refrigerating the expressed breast milk within 1 h of expression, and freezing within 24 h if it was not possible to bring in the milk within 48 h to hospital [37]. The Monash guideline also recommended to freeze the expressed breast milk if it will not be transported within 24 h [24]. Similar to MHW, the RWH guideline recommended frozen milk that was partially thawed on arrival to be thawed completely in the refrigerator and be used within 24 h [20, 37].

There were no Sri Lankan recommendations on transportation of expressed breast milk.

#### Other information

The Sri Lankan pamphlet mentioned that there is no difference in the taste or goodness of expressed breast milk (which is used fresh in Sri Lanka) versus milk obtained by direct breastfeeding [32]. This pamphlet also mentioned not to use bottles with teats for feeding the milk and to use a cup or spoon instead.

## Discussion

The purpose of this paper was to review selected guidelines and factsheets on expression and storage of breast milk, both at home and in the neonatal unit, and on transport of expressed breast milk from home to hospital, in order to assist in establishing safe standards for transporting expressed breast milk in Sri Lanka from home to neonatal units in the hospital for mothers who are unable to stay in hospital with their sick newborns. In reviewing the selected guidelines and fact sheets we noted that most recommendations on general aspects of breast milk expression, how to hand-express and freezer storage guidelines were similar in the different guidelines. However, when taking each information source individually there were gaps, wide variations and unclear areas with regard to the method of transport. There is therefore a need for a written single guideline, for each unit which contains recommendations on all aspects of expressing, storing and transporting breast milk which has the same basic information for mothers and the healthcare staff and further technical details for staff if required. The Sri Lankan fact sheet strongly discourages the use of pumps, even going to the extent of stating that it is more painful. Concerns about cleanliness have also contributed to the discouragement [32]. In Sri Lanka – especially for hospital based use, only fresh expressed breast milk is generally used. Therefore, currently there is no necessity for expressing large volumes for storage in Sri Lanka. This may be the reason for discouragement of pump use along with concerns about the cost of pumps as well – although hand pumps are now available for very reasonable prices. The available guidelines have been written nearly a decade ago and neonatal care, especially in terms of survival of preterm infants has improved greatly since then. Therefore, the Sri Lankan information sheet needs to be updated with more evidence-based recommendations that are relevant to the current situation of sick newborns in the country. There is an urgent need to identify safe modes of storage and transport of expressed breast milk in Sri Lanka, taking into consideration available modes of storage and transport along with weather conditions. Sri Lanka is an island situated within the tropics where the mean annual temperature varies between 27 °C in the coastal lowlands to 16 °C in the central highlands. Even in the highlands the maximum daytime temperatures are more than 18.5 °C [38]. The average relative humidity is > 65% in all parts of the country and above 75%, up to 95%, in the wet zone [39].

The Sri Lankan fact sheet does not mention the use of photographs of the baby to stimulate hormonal responses in the mother because it is currently not relevant as mothers will be doing most of the expression of breast milk in the neonatal unit itself [32]. However, it would be very useful for the mothers who are unwell in intensive care unit themselves and therefore may not even have seen the baby yet. There are hospital regulations in Sri Lanka which prohibit photography of patients which would need to be addressed. Other methods of stimulating a hormonal response which enhance milk secretion, that could be mentioned in a guideline or fact sheet for mothers include kangaroo mother care and back massage for the mothers [31].

With regard to containers, the Sri Lankan recommendation is the use of wide-mouthed containers as they can then be used directly for cup-feeding of the baby. In Sri Lanka, in keeping with the ten steps of the Baby Friendly Hospital Initiative, the recommended method of feeding expressed breast milk even at home is by cup or rarely spoon; mothers are advised to avoid teats and bottles for feeding the expressed breast milk; it is always cups or spoons that are used for feeding of supplementary expressed breast milk even after the babies are discharged home. This recommendation should be considered by other institutes worldwide as well, if they are hoping to achieve baby-friendly hospital status.

This paper is the first component of a series of studies to establish the necessity and safety of an economical method of expressing, storing and transporting breast milk in Sri Lanka from home to hospital, for mothers who are unable to stay in hospital for a prolonged period with their sick newborns. A Hazard Analysis and Critical Control Points is a system designed to ensure food safety by preventing hazards due to microbiological contamination, biochemical and physical changes that occur in food items from the stage of raw material to the finished product that would be consumed and a previous study in Belgium has studied this in 2011 for expressed breast milk on a neonatal unit [8]. When the final version of the recommendations for Sri Lanka are prepared, the points that need to be addressed e.g. method of hand expression including cleaning of hands before expression of milk, type of container used for storage, cleansing of the container for storage, methods of storage and transportation of the expressed breast milk that will maintain desired temperatures and acceptable microbiological status, will be identified using the guidelines and protocols that have been studied in this paper, taking economical and sociocultural aspects of Sri Lanka into consideration. The availability of a refrigerator or freezer at home and transport modes that will be used by mothers or the person bringing in milk from home to hospital will be studied prior to making any recommendations. The guideline we prepare will include a section on breastfeeding counselling and supporting a mother to build her self-confidence.

## Conclusion

A single document containing recommendations on all aspects of expressing, storing and transporting breast milk should be available for each unit, with the same basic information for mothers and the healthcare staff and further technical details for staff if required. The Sri Lankan recommendations need to be updated based on current worldwide practices and further studies are needed to establish a safe method of transport of expressed breast milk in Sri Lanka.

## Additional files

Additional file 1: **Table S1.** General information on expression of breast milk and preparation for expression; tabulation of recommendations from the different institutions (XLSX 11 kb)

Additional file 2: **Table S2.** Container for collection and storage of expressed breast milk; tabulation of recommendations from the different institutions (XLSX 10 kb)

Additional file 3: **Table S3.** Container for collection and storage of expressed breast milk; tabulation of recommendations from the different institutions (XLSX 11 kb)

Additional file 4: **Table S4.** Container for collection and storage of expressed breast milk; tabulation of recommendations from the different institutions (XLSX 10 kb)

Additional file 5: **Table S5.** Container for collection and storage of expressed breast milk; tabulation of recommendations from the different institutions (XLSX 11 kb)

Additional file 6: **Table S6.** Container for collection and storage of expressed breast milk; tabulation of recommendations from the different institutions (XLSX 11 kb)

Additional file 7: **Table S7.** Container for collection and storage of expressed breast milk; tabulation of recommendations from the different institutions (XLSX 10 kb)

## Abbreviations

AGREE-II: Appraisal of Guidelines for Research & Evaluation – II
HMBANA: Human Milk Banking Association of North America
MHW: Mercy Hospital for Women, Melbourne, Australia
Monash: Monash Melbourne, Australia
NHS: National Health Service, UK webpages
RCH: Royal Children’s Hospital, Melbourne, Australia
RWH: Royal Women’s Hospital, Melbourne, Australia
SL: Sri Lanka
WHO/UNICEF: World Health Organization / United Nations Children’s Emergency Fund
